# Solvent-Free One-Pot Synthesis of Epoxy Nanocomposites Containing Mg(OH)_2_ Nanocrystal–Nanoparticle Formation Mechanism

**DOI:** 10.1021/acs.langmuir.2c00377

**Published:** 2022-04-28

**Authors:** Francesco Branda, Jessica Passaro, Robin Pauer, Sabyasachi Gaan, Aurelio Bifulco

**Affiliations:** † Department of Chemical Materials and Industrial Production Engineering (DICMaPI), University of Naples Federico II, 80125 Naples, Italy; ‡ Advanced Materials and Surfaces Fibers, Empa Swiss Federal Laboratories for Materials Science and Technology, CH-8600 Dubendorf, Switzerland; § Laboratory for Advanced Fibers, Empa Swiss Federal Laboratories for Materials Science and Technology, Lerchenfeldstrasse 5, 9014 St. Gallen, Switzerland

## Abstract

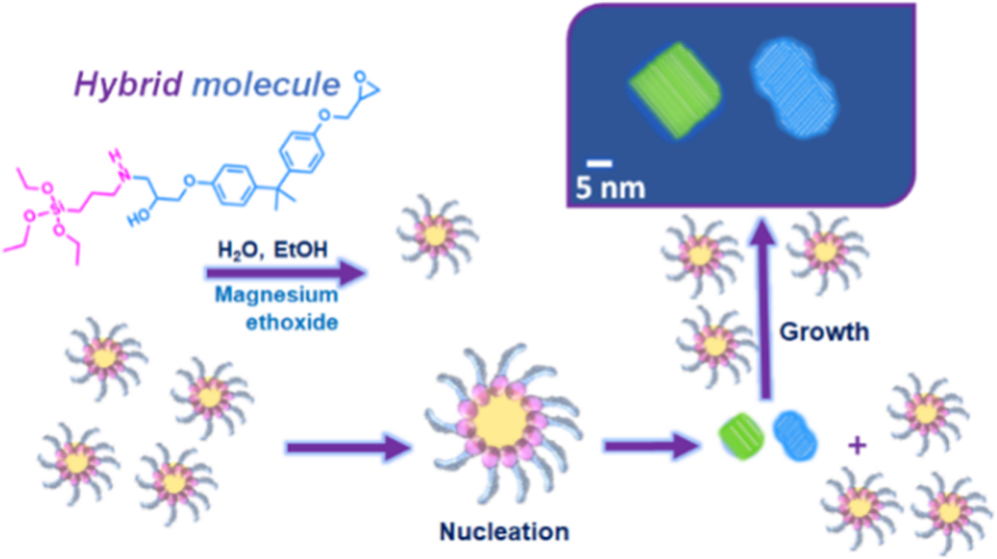

Epoxy nanocomposites containing Mg(OH)_2_ nanocrystals (MgNCs, 5.3 wt %) were produced via an eco-friendly “solvent-free one-pot” process. X-ray diffraction (XRD), high-resolution transmission electron microscopy (HRTEM), and thermogravimetric analysis (TGA) confirm the presence of well-dispersed MgNCs. HRTEM reveals the presence also of multisheet-silica-based nanoparticles and a tendency of MgNCs to intergrow, leading to complex nanometric structures with an intersheet size of ∼0.43 nm, which is in agreement with the lattice spacing of the Mg(OH)_2_ (001) planes. The synthesis of MgNCs was designed on the basis of a mechanism initially proposed for the preparation of multisheet-silica-based/epoxy nanocomposites. The successful “in situ” generation of MgNCs in the epoxy via a “solvent-free one-pot” process confirms the validity of the earlier disclosed mechanism and thus opens up possibilities of new NCs with different fillers and polymer matrix. The condition would be the availability of a nanoparticle precursor soluble in the hydrophobic resin, giving the desired phase through hydrolysis and polycondensation.

## 1 Introduction

Magnesium hydroxide is popular in applications spanning different fields such as wastewater treatment,^1,2^ flue gases treatment in environment protection,^2,3^ stability and release improvement of poly(lactic-*co*-glycolic) acid (PLGA)-based injectable implant delivery system for the controlled release of proteins,^4^ wood pulp bleaching in pulp and papermaking industry,^5,6^ ethanol chemical sensor,^7^ antibacterial and antifouling applications,^8−11^ and flame retardant.^12−19^ It has also been proposed as a precursor of MgO crystals that retain crystallite size and morphological features of the parent phase.^2,6,19−22^

Mg(OH)_2_ with different shapes, such as nanorods, fibers, nanotubes, nanoflakes, nanoparticles, nanosheets, nanoplates, and nano- and microdisks were successfully obtained following various approaches.^6,22^ The resulting morphology is strictly dependent not only on the production methodology but also on the specific conditions adopted for their synthesis.^6,22^ Many Mg(OH)_2_ nanoparticles preparation methods, including precipitation, hydrothermal, and solvothermal as well as sol–gel, have been developed, although most of them are at laboratory scale.^2,6,22^ Among the numerous applications of Mg(OH)_2_ listed above, flame-retardant applications to develop halogen-free flame retarding (HFFR) polymers are very important thanks to its nontoxicity,^2,12−18,23,24^ ability to suppress smoke, and tendency to decompose to give magnesium oxide and CO_2_. Its flame-retardant action is reported to be strictly linked to decomposition during polymer combustion.^24,25^ In fact, the decomposition absorbs heat, produces gas that dilutes the flame, and causes MgO particles to behave as a thermal shield and an oxygen barrier.

Mg(OH)_2_ is highly hydrophilic and thus surface treatments with silane (very popular is γ-aminopropyltriethoxysilane (APTES)) are necessary to improve dispersion in hydrophobic matrices like epoxy or polypropylene.^18,25,26^ It is also known that the mechanical reinforcing effect and, often, the flame-retardant efficiency of magnesium hydroxide increase when it is introduced as nanosized instead of microsized crystals.^12,27^ This is an example of the reasons why organic/inorganic hybrid (HOI) and nanostructured composites have received so much attention recently.^28−40^ Very often, their synthesis gave unprecedented materials whose properties are not simply a combination of the two components alone.^41^

This paper proposes a novel “solvent-free one-pot” process that results in epoxy nanocomposites containing Mg(OH)_2_ nanocrystals. It is worth reminding that much of the recently published literature is devoted to the “in situ” synthesis of silica nanoparticles in the epoxy matrix.^42−55^ Many of them explored the solvent-free one-pot route, which appears to be particularly interesting. It is worth reminding, in fact, that production and functionalization of particles require the use of solvents that must be subsequently removed, recycled, and/or disposed of, with environmental, health, safety, and cost issues. Further, the preparation of nanocomposites by solution blending involves a complicated manufacturing process including synthesis of nanoparticles, surface functionalization, high pressure and temperature mixing with polymer resins, and solvent evaporation. The solvent-free one-pot synthesis process encompasses nanosilica formation and functionalization (without solvent aid) and nanocomposite hardening in one pot with high silica dispersion and strong silica–epoxy adhesion simultaneously achieved in an eco-friendly manner. The silica nanoparticles are obtained through the sol–gel synthesis in the presence of epoxy resin. In the case of tetraethoxysilane, this involves the following reactions:^56−58^
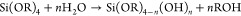
1
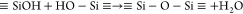
2
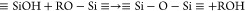
3In some literature, epoxy resin functionalized with ethoxy groups, obtained through the reaction of epoxy with γ-aminopropyltriethoxysilane ((H_2_N(CH_2_)_3_Si(OC_2_H_5_)_3_), APTES), was also used as a silica precursor.^43^ However, APTES was generally added as an essential coupling agent for nanosilica, necessary to obtain good dispersion and tailoring of the organic/inorganic interphase, as reported in the literature,^43−55^ also for very unusual flame-retardant fillers like the humic acids.^59^

The authors,^60,61^ recently, followed an approach very close to the solvent-free one-pot one proposed by Jiao^50^ to obtain silica/epoxy nanocomposites. Although the content was very low, the well-dispersed nanosized silica produced through the in situ methodology had a strong beneficial effect on the fire behavior that often prevents composite applications because of severe regulations (i.e., in aerospace engineering).^60,62−64^ High-resolution transmission electron microscopy (HRTEM) and small- and wide-angle X-ray scattering (SAXS/WAXS) performed by means of a multirange device Ganesha 300 XL+ allowed us to have an insight into the structure of nanoparticles.^61^ It was possible to recognize the multisheet structure of the silica-based nanoparticles and to propose a mechanism for their formation.^61^

In this paper, it is shown that, on the basis of the proposed mechanism, the formation, in epoxy (or other hydrophobic resins), of nanoparticles of different chemical nature other than silica may be foreseen. It is recognized that a nanoparticle precursor needs to satisfy the following two conditions: solubility in the hydrophobic resin and the ability to give the new phase through hydrolysis and, possibly, polycondensation. In this prospect, in this work we have synthesized magnesium hydroxide nanoparticles in epoxy, using magnesium ethoxide (Mg(OC_2_H_5_)_2_) as the precursor that satisfies the defined conditions. X-ray powder diffraction, microscopy, and thermogravimetric analysis (TGA) were used to prove the formation of Mg(OH)_2_ nanoparticles. The results, while confirming the validity of the mechanism, prove that the solvent-free one-pot approach may be successfully extended for the preparation of hybrid nanocomposite systems other than the silica–epoxy-based ones.

## 2 Experimental Section

### 2.1 Materials

Tetraethyl orthosilicate (TEOS, >99%), (3-aminopropyl)-triethoxysilane (APTES, >98%), magnesium ethoxide (>98%), and ethanol (ACS reagent, anhydrous) were purchased from Sigma-Aldrich (Switzerland). A two-component epoxy resin system (SX10 by MATES S.r.l., Milan, Italy), consisting of bisphenol A diglycidyl ether (DGEBA) and modified cycloaliphatic polyamines, was used for fabricating composite laminates.

### 2.2 Synthesis and Preparation of Epoxy/Mg(OH)_2_ Nanocomposite

The synthesis involved the following steps: (i) a mixture of 20 g of DGEBA and 5.9 g of magnesium ethoxide was stirred vigorously overnight at 80 °C; (ii) a mixture of 20 g of DGEBA and 3.5 g of APTES was stirred vigorously at 80 °C for 2 h; (iii) the second mixture was added to the first one and stirred vigorously at 80 °C for 30 min; (iv) distilled water (3.40 mL) and ethanol (1.08 mL) were added to the main batch and stirred vigorously at 80 °C under reflux for 90 min. The reaction vessel was, then, opened and kept at 80 °C for 30 min to remove ethanol and water; and (v) 10.4 g of hardener needed for curing was then added to the mixture at room temperature and mixed for 5 min. The resulting mixtures were degassed under vacuum and poured into a Teflon mold. The curing process was carried out at 30 °C for 24 h; then, the curing was completed by treating the samples at 80 °C for 4 h. The magnesium hydroxide content estimated from the stoichiometry was 5.3 wt %.

### 2.3 Characterization and Investigation Techniques

X-ray diffraction (XRD) measurements were performed using a Philips X’Pert-Pro diffractometer using monochromatic Cu Kα radiation (40 mA, 40 kV) with a step width of 0.013° (2θ). JCPDS cards were used to identify the crystalline phases. Transmission electron microscopy (TEM) images of composite samples were recorded using a TEM/STEM JEOL JEM 2200 fs microscope operating at 200 kV. Prior to TEM analysis, powders of the sample were prepared and dispersed in water and a drop of the finely dispersed sample was put on a Lacey Carbon film copper TEM grid. The TEM grid with the sample droplet was dried overnight in an oven at 40 °C. Fifty particles at random locations were analyzed by Image J to determine the particle size and distribution. HRTEM images were used to determine the lattice plane distance using the Image J software. Thermogravimetric analysis (TGA) was carried out to study the thermal behavior of the prepared materials using a Q500 system from TA Instrument (New Castle, DE); the samples were heated from 50 to 700 °C at 10 °C/min in nitrogen or air (gas flow of 60 mL/min). The tests were performed by placing about 10 mg of the sample in open alumina pans.

## 3 Results and Discussion

### 3.1 Synthesis Design

**Scheme 1:**
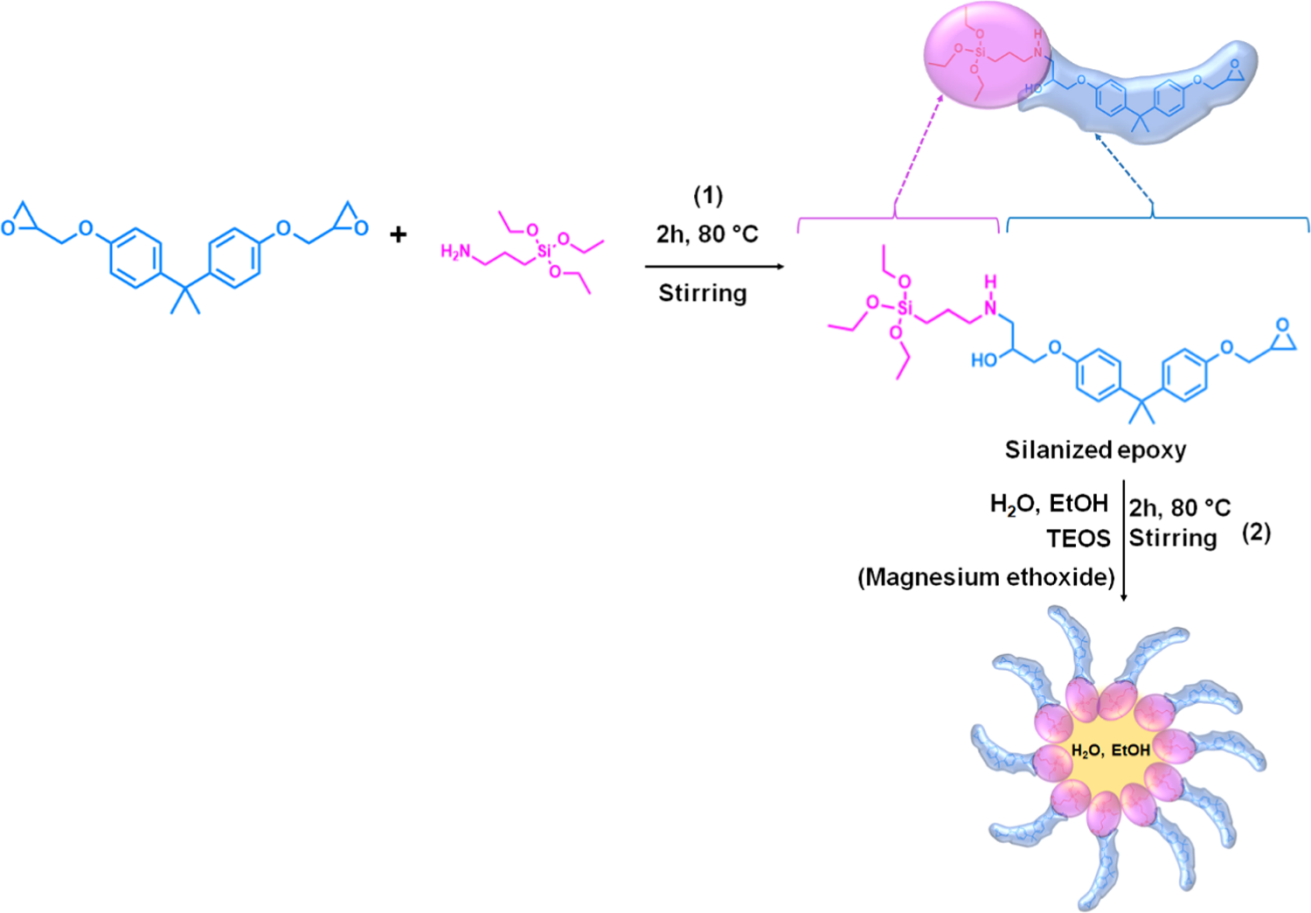
Formation Mechanism of the Silanized Epoxy Hybrid in Step 1 and Mg(OH)_2_ Nanoparticles in Step 2.

Recently, silica-based nanoparticles have been produced in situ in an epoxy matrix following a procedure similar to the one already reported in the literature.^50,60^ HRTEM and the combined small- and wide-angle X-ray scattering (SAXS/WAXS) help prove a multisheet structure for the silica nanoparticles.^61^ A mechanism was proposed for their in situ formation based on the concepts of the surfactant-aided sol–gel chemistry,^61^ which recently allowed us to obtain new mesoporous gel textures with very interesting applications.^65−70^ The sol–gel synthesis of silica nanoparticles was performed in the presence of epoxy resin and very limited amounts of water and ethanol.^61^ TEOS and APTES were used as silica precursors. However, first, APTES was added to epoxy resin and left to react with it to obtain a silanized epoxy molecule as represented in the first step of Scheme 1(50,60,61)

It is hypothesized that^61^ upon addition of TEOS water and alcohol, the hybrid molecule plays the role of a surfactant as shown in the second step of Scheme 1. In fact, on one hand, it exposes three ethoxy groups that may be hydrolyzed (see reaction 1), which makes them hydrophilic; on the other hand, it exposes the DGEBA molecule that causes it to be strongly “epoxyphilic”. Therefore, micelles are hypothesized to form in the epoxy matrix by collecting the added water and ethanol.^61^ These micelles constitute the nanoenvironment where, owing to the very high water/TEOS weight ratio, the precursor of silica, initially dissolved in epoxy, may be fully hydrolyzed and polycondensate to silica-based oligomers through the reactions described by eqs 1–3. The crystalline structures observed in HRTEM are formed through a mechanism of nucleation and crystal growth similar to the one initially proposed by Tamman exploiting the formed micelles,^71−74^ as previously reported.^61^

On the basis of this mechanism, we may foresee the in situ formation of phases that are of very different nature than silica as long as a precursor is soluble in the hydrophobic epoxy resin and able to give the desired phase through reactions of hydrolysis and, possibly, polycondensation does exist. This is the case of Mg(OH)_2_ nanocrystals that may be obtained by the hydrolysis of Mg(OC_2_H_5_)_2_, which is found to be soluble in epoxy resin under stirring at 80 °C. We may suppose that in this case also the micelles may play the role of suitable nanoenvironment for the production of the “small structural units” needed for the nucleation and growth of Mg(OH)_2_ nanocrystals. Therefore, magnesium ethoxide is added to Scheme 1 in parenthesis: it represents the alternative precursor to obtain Mg(OH)_2_ instead of silica-based nanoparticles. A synthesis path very close to the one previously followed for silica/epoxy is explored.^50,60,61^ Dissolution of magnesium ethoxide in epoxy at 80 °C, however, is a very slow process. This is the reason why, as described in the Experimental Section, we separately dissolved magnesium ethoxide and APTES in epoxy. Afterward, the two solutions were mixed and nanocomposite curing was performed in one pot after the formation of both nanoparticles.

### 3.2 Morphological Analysis and Composition Study of the Nanocomposite

**Figure 1:**
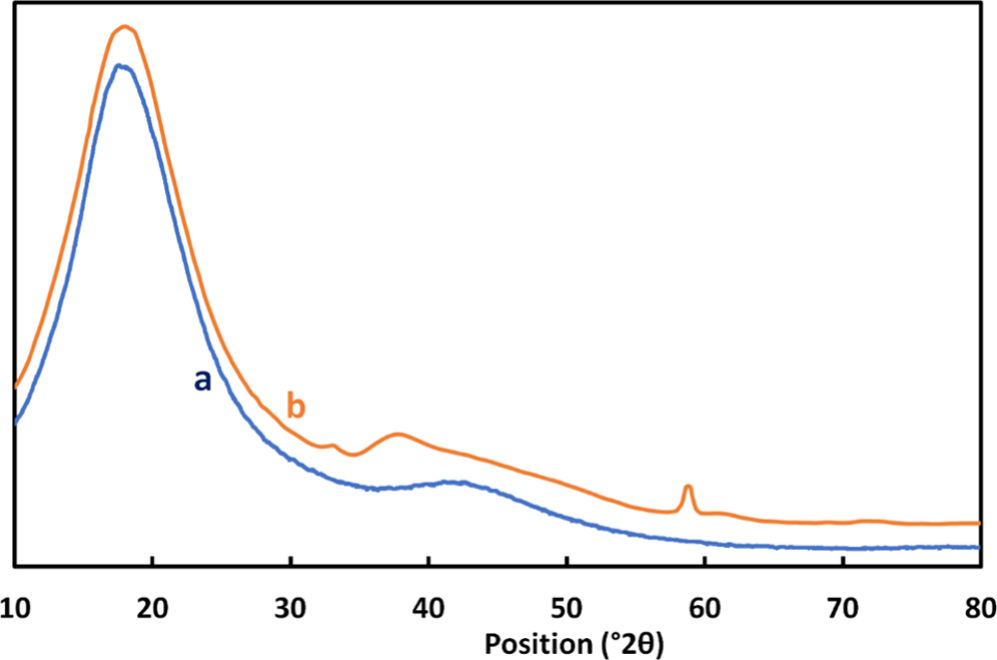
XRD patterns of neat epoxy (a) and nanocomposite (b).

Figure S1 shows the photograph of a nanocomposite sample. The transparency suggests that very well dispersed very tiny particles of Mg(OH)_2_ are present in the matrix. Figure 1 shows the X-ray diffraction (XRD) patterns of neat epoxy and Mg(OH)_2_ nanocomposite. As can be seen, the (101) and (110) peaks (2θ = 38.41 and 59.03°) of the hexagonal Brucite structure of Mg(OH)_2,_ (JCPDS 7-239)^19,75,76^ are clearly visible in the nanocomposite pattern, thus supporting the formation of magnesium hydroxide. The significant peak broadening indicates that Mg(OH)_2_ has a very small grain size. After analyzing the XRD spectra for all of the investigated samples, we applied the Scherrer equation considering the main peaks for the crystalline phase. The authors carried out a procedure reported in the literature,^77,78^ which is same as that used in a previous work dealing with the surface modification of magnesium hydroxide and its application in flame-retardant polypropylene composites. The authors show that the average size for the crystallites is 101.4 nm.

**Figure 2:**
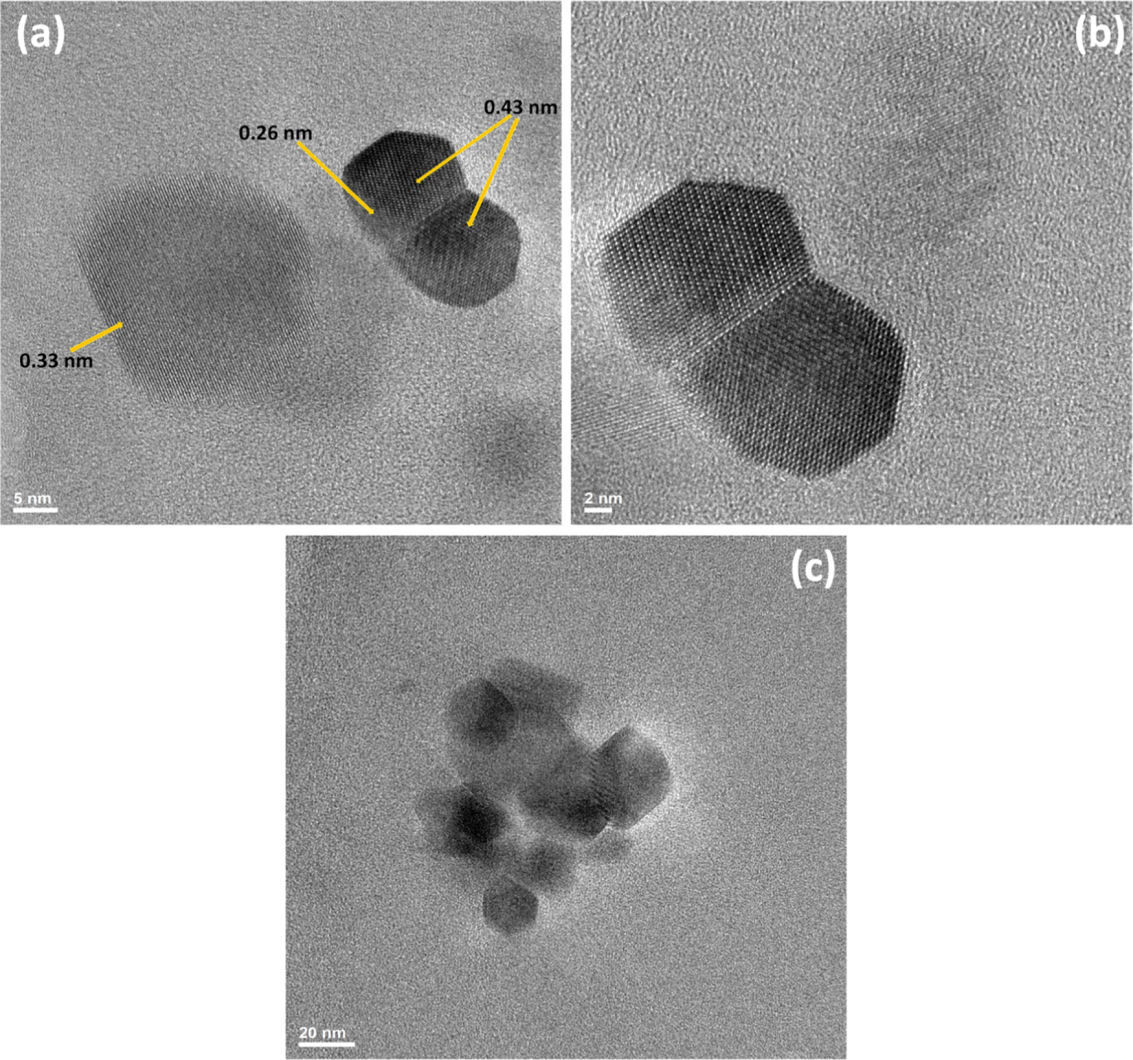
HRTEM of (a) nanocomposite, (b) part of (a), and (c) larger-sized crystals resulting from the tendency of Mg(OH)_2_ nanocrystals to intergrow.

Figure 2a shows two typical particles that are observed in HRTEM. It confirms that the particles are of nano size as suggested by the nanocomposite’s transparency and XRD reflection shape. As better shown by the micrograph at a greater enlargement (Figure 2b), two twinned crystals on the right side possess the pseudo-hexagonal morphology reported for Mg(OH)_2_ crystals.^6^ In fact, Figure 2b clearly shows that the two crystals share some of the same crystal lattice points in a symmetrical manner. The intersheet distance is measured as shown in Figure S2 and reported in a previous paper.^61^ As indicated in Figure 2a, two intersheet sizes were 0.43 and 0.26 nm. The first one agrees well with the lattice spacing of the Mg(OH)_2_ (001) planes.^6^ The second one may be attributed to the (111) crystal plane of periclase (MgO), which may be derived from Mg(OH)_2_ when exposed to an electron beam as already reported.^6^ Otherwise, the exposition to the beam of an electron microscope is proposed as a way to obtain MgO crystals.^79,80^Figure 2a shows also (on the left side) the second nanoparticle of similar size but different morphology than the one on the right. The morphology is very similar to that of the multisheet-silica-based nanoparticles described in the previous paper.^61^ The intersheet distance of 0.32 nm is also close to the one reported therein. The presence of such kind of nanoparticles may be explained due to the presence of APTES. Its addition was intended as the coupling agent. However, APTES is also by itself a good precursor of silica-based nanoparticles.^81^ Otherwise, in the proposed mechanism,^61^ the multisheet structure of the silica-based nanoparticles was just explained through the involvement of APTES in the hydrolysis and polycondensation reaction leading to their formation. Figure S3 shows another example of the great tendency of the intergrowth of Mg(OH)_2_ nanocrystals, already reported elsewhere.^6^ Also, in this case, the intersheet distance is 0.45 nm. Larger size crystals are also observed, as shown in Figure 2c. The complex structure of some of them appears to be the result of the strong tendency to intergrow.

It is worth pointing out that the size distribution is different from that of silica sheet nanoparticles reported in our previous paper.^61^ In that case, according to the small-angle X-ray scattering (SAXS) results, HRTEM micrographs show that the size of particles range from a few nanometers to a maximum of 30 nm. In the present case, the average size of the crystallite calculated by the Scherrer formula is 101.4 nm and the size distribution is much broader.

### 3.3 Thermogravimetric Analysis of the Nanocomposite

**Figure 3:**
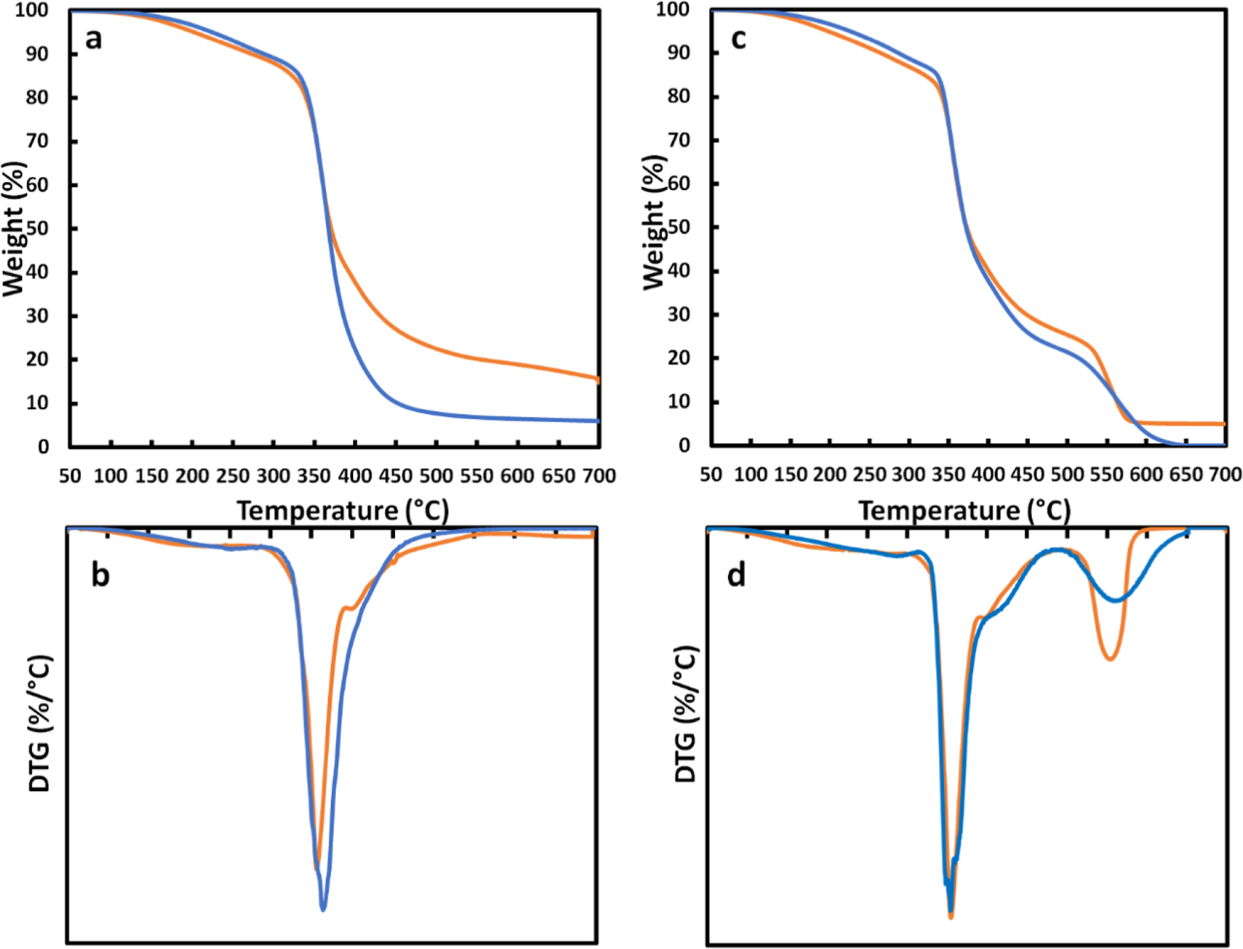
(a, b) TGA curves and DTG curves of EPO (blue) and EPO_Mg(OH)_2_ (orange) under nitrogen. (c, d) TGA curves and DTG curves of EPO (blue) and EPO_Mg(OH)_2_ (orange) under air.

Figure 3a,b shows the thermograms recorded in nitrogen atmosphere for epoxy and nanocomposite (Figure 3a) and their derivative curves (DTG) (Figure 3b). Figure 3c,d shows similar curves recorded in air. We obtained the typical TGA curves reported in the literature.^82−84^ As reported in the literature, unlike only one stage of reaction in the inert atmosphere, two reaction stages are involved when oxygen is present as the carrier gas. The degradation mechanisms are different in the two cases. Epoxy resin is a charring polymer that undergoes pyrolysis in nitrogen to mostly provide abundant aliphatic char around 350–400 °C, whereas the presence of oxygen triggers thermo-oxidative degradation, which leads to the formation of an aromatic char at ca. 600 °C. Therefore, in this case, the highest weight loss occurs in the two steps and later in the decomposition process.^85−87^

A shoulder (well evidenced as a peak of the DTG curve) is superimposed with the degradation curve of the nanocomposite. The decomposition peak temperature occurs at 398 °C (Figure 3b). It is worth reminding that pronounced weight loss, due to Mg(OH)_2_ decomposition, is reported to occur in the temperature range of 280–450 °C with a maximum decomposition temperature at 352 °C.^75,76^ Therefore, TGA results are in good agreement with the presence of magnesium hydroxide. When performing TGA in the air (curves 3c), the typical TGA curves of epoxy are obtained with oxidative degradation occurring in two steps. In this case, a very low (0.3 wt %) final residue was recorded for epoxy. Instead, in the case of nanocomposite, the amount of residue of 5.0 wt % is close to the one expected by taking into account that the MgO obtained from Mg(OH)_2_ is estimated to be 3.6 wt % and the silica-based phase estimated from the APTES content is close to 1.8 wt %. Therefore, the amount of residue, 5.0 wt %, closely corresponds to the expected one.

Finally, it is worth pointing out that the synthesis route proposed in the present paper is radically different from the other interesting ones recently proposed in the literature.^88^ The present paper shows the application of a “solvent-free one-pot” method in which the nanoparticles are produced through in situ sol–gel chemistry. The water and ethanol amounts added are minimal: water is added to the epoxy in a ratio of 3.40/40 g and ethanol in the ratio of 1.08 mL/40 g. Their additions in limited amount are intended at the sol–gel reactions. In fact, reactions 1 and 3 show that (a) water is the reagent of the hydrolysis reaction and (b) ethanol is the product of the hydrolysis and polycondensation reactions of the sol–gel method; therefore, its concentration affects the reaction evolution.^56−58^ On the contrary, in the case of the above-mentioned paper,^88^ 1.0 g of magnesium acetate and 5 g of epoxy are dissolved in 8 mL of methanol and 10 mL of acetone and mixed with an aqueous solution of NaOH (2g in 100 mL). Therefore, it is not about a “solvent-free” process. The procedure is even not a “one-pot” one; it requires operations of vacuum filtration, washing, and drying. Moreover, the present procedure allows us to avoid the use of acetone, which is known to adversely affect some important composite properties like glass transformation temperature.^89^

It is also worth pointing out that in the first attempts, APTES was not added to the synthesis batches. Although the final expected MgOH_2_ content was only 2%, it was not possible to produce transparent samples. This well proves the importance of silanized epoxy hybrid in the formation of Mg(OH)_2_ nanoparticles.

## 4 Conclusions

XRD, HRTEM, and TGA results prove that epoxy nanocomposites containing well-dispersed Mg(OH)_2_ nanocrystals (5.3 wt %) were successfully produced through the proposed solvent-free one-pot process. HRTEM reveals a great tendency of Mg(OH)_2_ nanocrystals to intergrow, giving rise to nanometric structures where the intersheet size is about 0.43 nm, well in agreement with the lattice spacing of the Mg(OH)_2_ (001) planes. HRTEM shows also the presence of multisheet nanoparticles similar to the silica-based ones reported in a recent paper by the authors. Both nanoparticles were produced in situ in the presence of epoxy resin through a procedure similar to the one described in the same paper. According to the mechanism therein proposed, it was simply necessary to substitute one of the silica precursors (TEOS) with Mg(OC_2_H_5_)_2_.

The results, while confirming the validity of the mechanism, prove that, at its base, nanocomposites of different chemical nature in addition to the well-established silica/epoxy composites can be produced through a solvent-free one-pot process provided proper particles precursors are available. The prerequisite is that the precursor must be soluble in the hydrophobic resin and able to give the desired phase through reactions of hydrolysis and, possibly (as in the case of silica-based particles), polycondensation.

## Supporting Information Available

Photographs of nanocomposite sample; determination of lattice plane distance from HRTEM images of the nanocomposite using Image J software; and HRTEM of nanocomposite showing the tendency to intergrowth of Mg(OH)_2_ nanocrystals (PDF)
