# Supplementary material for: Solvent-Free One-Pot Synthesis of Epoxy Nanocomposites Containing Mg(OH)2 Nanocrystal–Nanoparticle Formation Mechanism
Source: Langmuir. 2022 Apr 28;38(18):5795–802. doi: 10.1021/acs.langmuir.2c00377 (PMC9097534; doi:10.1021/acs.langmuir.2c00377)
Supplement: Supplementary file 1 — la2c00377_si_001.pdf [file la2c00377_si_001.pdf]

Supporting Information

Solvent-Free One-Pot Synthesis of Epoxy Nanocomposites Containing $Mg(OH)_2$ Nanocrystals – Nanoparticles Formation Mechanism

Francesco Branda[1,], Jessica Passaro[1], Robin Pauer[2], Sabyasachi Gaan[3], Aurelio Bifulco[1]*

[1]Department of Chemical Materials and Industrial Production Engineering (DICMaPI) University of Naples Federico II, Naples, Italy

[2]Advanced Materials and Surfaces Fibers, Empa Swiss Federal Laboratories for Materials Science and Technology, CH-8600 Dubendorf, Switzerland

[3]Laboratory for Advanced Fibers, Empa Swiss Federal Laboratories for Materials Science and Technology, Lerchenfeldstrasse 5, 9014 St. Gallen, Switzerland

Corresponding Author

*E-mail: branda@unina.it

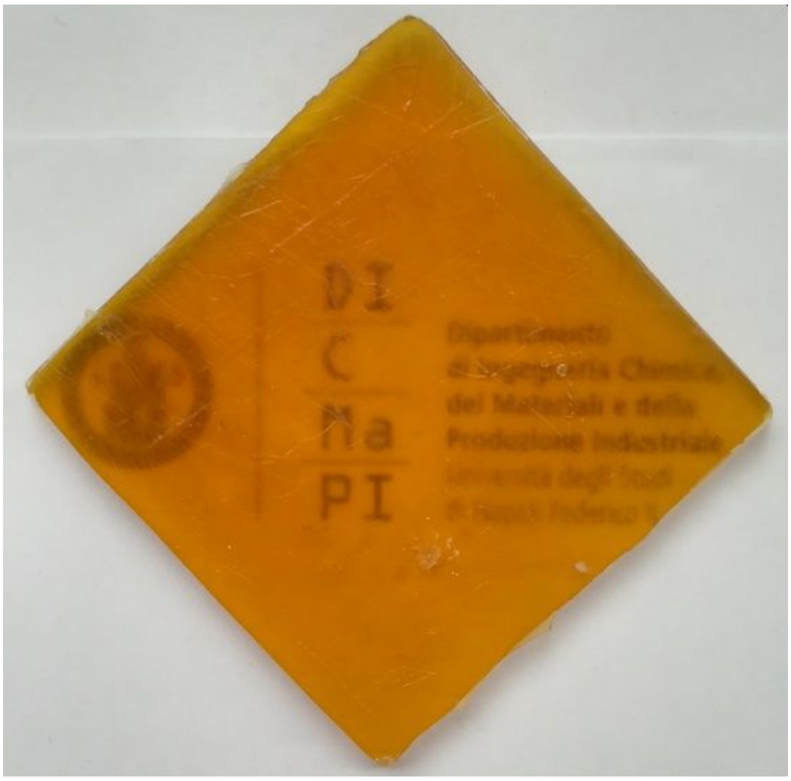

Figure S1. Photo of nanocomposite sample.

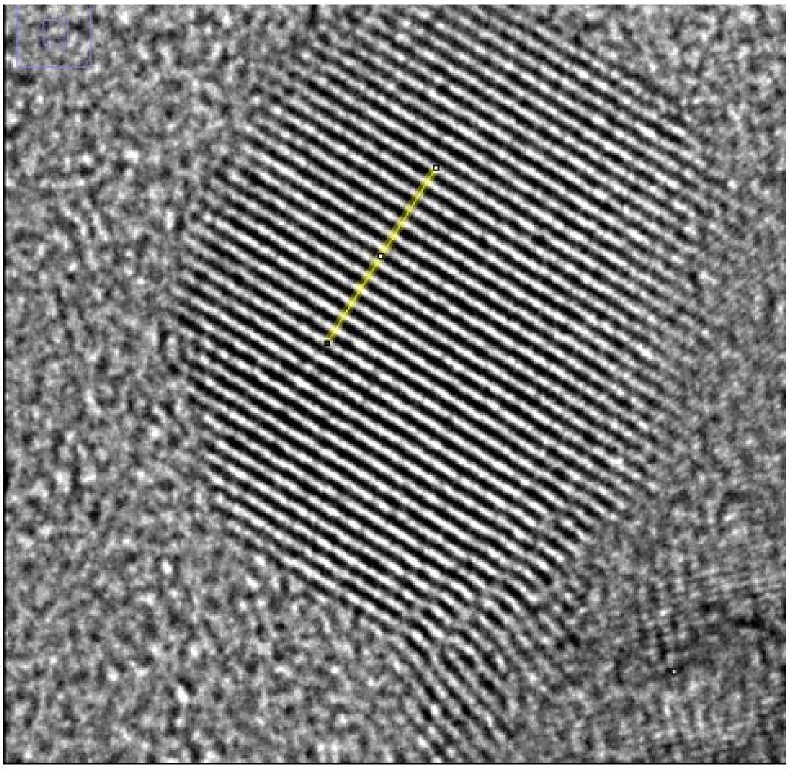

Figure S2. Determination of lattice plane distance from HRTEM images of the nanocomposite by using Image J software.

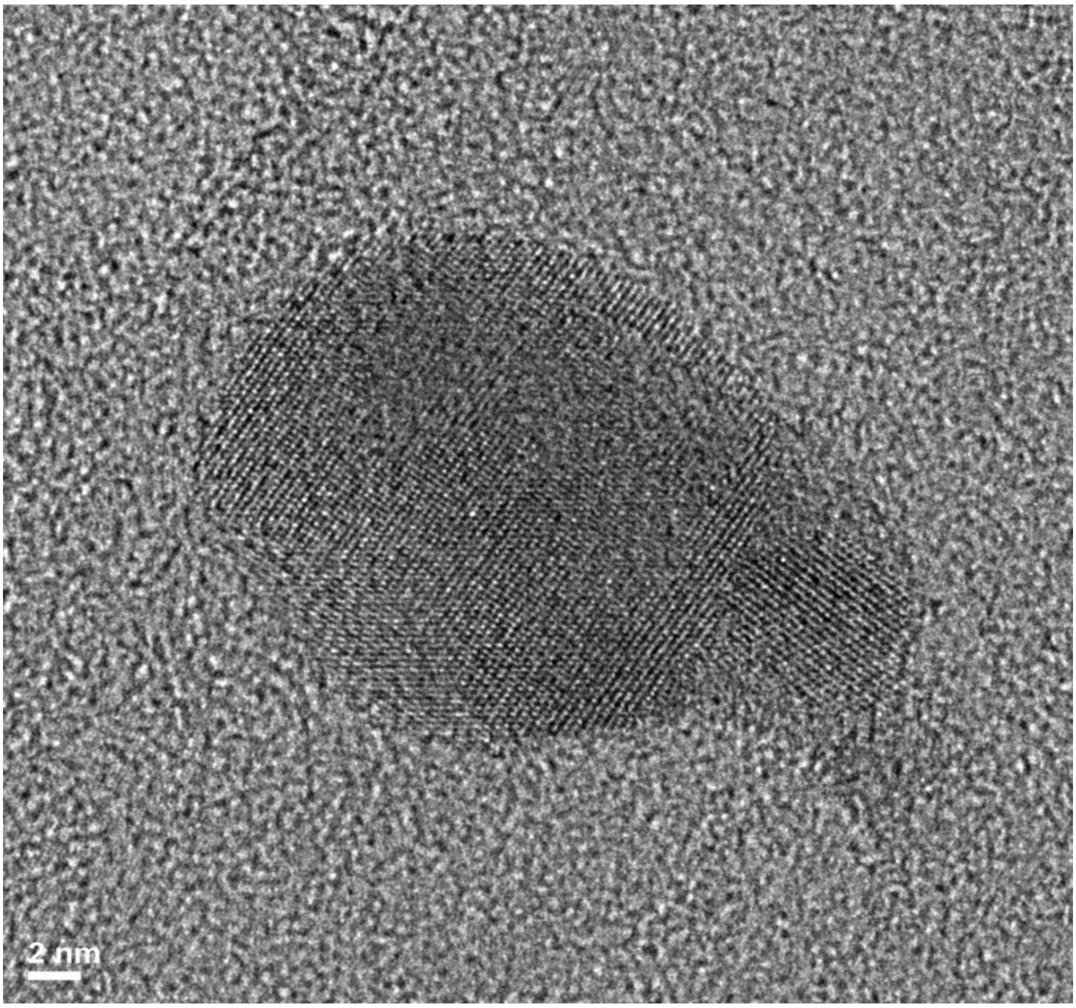

Figure S3. HRTEM of nanocomposite showing the tendency to intergrowth of $Mg(OH)_2$ nanocrystals.
